# Mutations identified in engineered *Escherichia coli* with a reduced genome

**DOI:** 10.3389/fmicb.2023.1189877

**Published:** 2023-05-25

**Authors:** Yuto Kotaka, Masayuki Hashimoto, Ken-ichi Lee, Jun-ichi Kato

**Affiliations:** ^1^Department of Biological Sciences, Graduate School of Science, Tokyo Metropolitan University, Tokyo, Japan; ^2^Department of Bacteriology I, National Institute of Infectious Diseases, Tokyo, Japan; ^3^Institute of Molecular Medicine, College of Medicine, National Cheng Kung University, Tainan, Taiwan

**Keywords:** *Escherichia coli*, genome-reduced strain, adaptive laboratory evolution, genome alteration, 3-phenylpropionate, oxidative stress

## Abstract

Characterizing genes that regulate cell growth and survival in model organisms is important for understanding higher organisms. Construction of strains harboring large deletions in the genome can provide insights into the genetic basis of cell growth compared with only studying wild-type strains. We have constructed a series of genome-reduced strains with deletions spanning approximately 38.9% of the *E. coli* chromosome. Strains were constructed by combining large deletions in chromosomal regions encoding nonessential gene groups. We also isolated strains Δ33b and Δ37c, whose growth was partially restored by adaptive laboratory evolution (ALE). Genome sequencing of nine strains, including those selected following ALE, identified the presence of several Single Nucleotide Variants (SNVs), insertions, deletions, and inversions. In addition to multiple SNVs, two insertions were identified in ALE strain Δ33b. The first was an insertion at the promoter region of *pntA*, which increased cognate gene expression. The second was an insertion sequence (IS) present in *sibE*, encoding the antitoxin in a toxin-antitoxin system, which decreased expression of *sibE*. 5 strains of Δ37c independently isolated following ALE harboring multiple SNVs and genetic rearrangements. Interestingly, a SNV was identified in the promoter region of *hcaT* in all five strains, which increased *hcaT* expression and, we predict, rescued the attenuated Δ37b growth. Experiments using defined deletion mutants suggested that *hcaT* encodes a 3-phenylpropionate transporter protein and is involved in survival during stationary phase under oxidative stress. This study is the first to document accumulation of mutations during construction of genome-reduced strains. Furthermore, isolation and analysis of strains derived from ALE in which the growth defect mediated by large chromosomal deletions was rescued identified novel genes involved in cell survival.

## 1. Introduction

Genome sequencing has facilitated the identification of essential genes and minimal gene sets for model micro-organisms such as yeast, *Bacillus subtilis*, and *Escherichia coli*. *Mycoplasma* encode a small genome, and subsequently all essential genes have been identified from the isolation and analysis of many transposon insertion mutants (Glass et al., 2006). A genome-reduced strain of *Mycoplasma* (JCVI-syn3.0) with a 531 kb genome approximately 49.2% the size of the wild-type genome was constructed, and the minimum gene set was experimentally characterized (Hutchison et al., 2016). This 473 gene set includes essential, quasi-essential, and nonessential genes, but the functions of 149 of these genes remain unknown (Hutchison et al., 2016). *E. coli* and *B. subtilis* have larger genomes than *Mycoplasma*, and all essential genes in these species were first identified by generating many gene-disrupted strains (Baba et al., 2006; Kato and Hashimoto, 2007). Functions of approximately 65.4% of all genes in *E. coli* have been experimentally characterized and those of only about 2.4% have not yet been estimated (Ghatak et al., 2019). Furthermore, the functions of almost all essential genes have been experimentally characterized (Kurata et al., 2015). Instead, it has not been possible to identify the minimum gene set by combining synthetic genes, as in studies of *Mycoplasma*. Genome-reduced strains have, however, been constructed by combining large-scale chromosomal deletions.

Genome-reduced strains of *E. coli* have been constructed using different strategies by several groups (Kurokawa and Ying, 2020). Blattner et al. compared the genomes of several *E. coli* strains with the goal of deleting genes that were introduced during evolution. Strain MDS43 was first constructed and harbors a deletion of approximately 15.27% of the wild-type genome (Pósfai et al., 2006). Strain MDS69 was later constructed by deleting foreign gene clusters identified through comparison of additional genomes and harbors deletion of 20.3% of the wild-type genome (Karcagi et al., 2016). Ogasawara et al. compared the genomes of *E. coli* with those of Buchnera spp., an insect symbiotic bacterium with a small genome and deleted chromosomal regions found only in *E. coli* to construct strain DGF-298, which lacks 36% of the wild-type genome (Hirokawa et al., 2013). An additional study described construction of strain MS56, which lacks 23% of the wild-type genome, by deletion of all ISs, K-islands, flagellar genes, ciliated genes, and lipopolysaccharide genes identified from the *E. coli* data bank (Park et al., 2014). *B. subtilis* strain PG38 strain, which harbors deletion of approximately 40% of the wild-type genome, has been described (Michalik et al., 2021).

We have previously constructed strains with global deletion mutations spanning the entire *E. coli* chromosome with the aim of identifying trans- and cis-acting genetic information essential for growth (Kato and Hashimoto, 2007). We subsequently identified that *oriC* is the only unique cis-acting genetic information (Kato and Hashimoto, 2007). We have also analyzed individual essential genes with unknown functions leading to identification of DNA topoisomerase IV, which is essential for chromosome segregation; Hda, which is essential for chromosomal replication initiation; and YqgF, which is essential for rRNA processing and so on (Kato et al., 1990; Kato and Katayama, 2001; Hashimoto et al., 2005; Kurata et al., 2015). By combining large-scale deletion mutations, we have constructed a series of genome-reduced strains (Δ1–Δ16) in which approximately 29.7% of the wild-strain chromosome was deleted and examined cell size, shape, and nucleoid organization (Hashimoto et al., 2005). We have also constructed a series of genome-reduced strains (Δ17–Δ33a) with approximately 38.9% deletion of the chromosome, examined their resistance to oxidative stress (Hashimoto et al., 2005; Iwadate et al., 2011), and identified genes involved in growth and survival. We later introduced large-scale chromosomal deletions into a series of genome-reduced strains, and by identifying genes displaying synthetic lethality, we identified a novel gene involved in DNA repair (Watanabe et al., 2016). Lastly, through analysis of a series of genome-reduced strains with differential resistances to the redox-cycling drugs menadione, we identified genes involved in oxidative stress resistance (Iwadate et al., 2017; Iwadate and Kato, 2017).

In this work, we constructed a series of genome-reduced strains (Δ33b-Δ41c) by introducing further deletions into the genome of strain Δ33a. During this process, we isolated strains whose growth rate was partially rescued by ALE. By sequencing nine strains, we clarified mutations commonly occurring during ALE and identified a novel gene involved in cell survival.

## 2. Materials and methods

### 2.1. Bacterial strains and culture media

All *E. coli* strains described in this study are derivatives of MG1655. Cells were grown in LB medium (1% Bacto tryptone, 0.5% Bacto yeast extract, and 1% NaCl) or Antibiotic Medium 3 (AM3, Becton Dickinson), unless otherwise stated. The approximate composition of AM3 is 1.5 gL^−1^ of beef extract, 1.5 gL^−1^ of yeast extract, 5 gL^−1^ of peptone, 1 gL^−1^ of dextrose, 3.5 gL^−1^ of sodium chloride, 3.68 gL^−1^ of dipotassium phosphate, and 1.32 gL^−1^ of monopotassium phosphate.

### 2.2. Construction of genome-reduced strains

Deletion mutants were constructed using the FRT4 system with some modifications (Iwadate et al., 2011, Supplementary Figure S1). Deletion mutants were constructed by introducing an Ap resistance cassette into the MG1655 *red* strain using the lambda phage red homologous recombination system. DNA fragments flanked by short regions of homology to chromosomal regions (25–40 bp) were prepared by PCR and introduced into cells by electroporation. Deletions were confirmed by PCR before preparation of P1 phage from the resulting cells. The Ap resistance gene was replaced with a Cm resistance gene by introducing Cm-FRT PCR fragment flanked with 25–40 bp sequences with homology to the Ap resistance gene. The positive selection marker was removed using a suicide vector encoding flanking sequences of the deleted region, an *rpsL*^+^ gene for negative selection, and the Ap resistance gene.

### 2.3. Isolation of ALE strains

The genome-reduced strain was aerobically cultured in 2 mL of AM3 medium until stationary phase. Cultures were subsequently diluted 1/20 with fresh medium, and the process was manually repeated until strains with increased growth were isolated.

### 2.4. Cell growth measurement

Genome-reduced strains were inoculated from an overnight culture and grown in AM3 at 37°C with vigorous shaking. Optical densities were measured and generation time calculated.

### 2.5. SNVs detection

Genomic DNA of *E. coli* was purified using a DNA extraction Kit (NucleoSpin Microbial DNA). Genomic DNA was enzymatically fragmented, and a DNA library was constructed using the Celero PCR Workflow and an Enzymatic Fragmentation DNA Seq Kit (TECAN) with a bead-based enrichment step to isolate library fragments greater than 400 bp. Paired-end sequencing was subsequently performed on the Illumina MiSeq system. Sequencing reads were processed using the CLC Genomics Workbench v10 (CLC Bio). Sequencing reads were trimmed of low-quality reads (limit = 0.1), ambiguous nucleotides, and adapter sequences (TruSeq universal and indexed adapter; GCTCTTCCGATCT). Trimmed reads were mapped to reference sequences with the following parameters: Minimum length fraction = 0.5, minimum similarity fraction = 0.8, match score = 1, mismatch cost = 2, insertion cost = 3, and deletion cost = 3. Sequences were considered variants if mutations were present in more than 50% of the mapped reads. The effects of variants were detected using SnpEff (Cingolani et al., 2012), and *rrn* variants were removed manually.

### 2.6. Construction and annotation of complete genomes

Genomic DNA for long-read sequencing was extracted using a KingFisher Duo Prime (Thermo Fisher Scientific) with the MagMAX DNA Multi-Sample Ultra 2.0 Kit. Sequencing libraries were prepared using a Rapid Barcoding Sequencing Kit (SQK-RBK004, Oxford Nanopore Technologies, Oxford, United Kingdom). A MinION R9.4.1 flow cell (Oxford Nanopore) was used for 48 h sequencing. Base calling was performed using the ‘super accurate’ algorithm. Long- and short-read sequences were subject to hybrid assembly by using Unicycler v.0.4.8 (Wick et al., 2017). Annotation of the complete genomes was performed using DFAST (Tanizawa et al., 2018) with manual curation.

### 2.7. Visualization of genomic changes

Complete genomes from short- and long-read hybrid assemblies were compared by BLAST (Altschul et al., 1990). Results were visualized using Kablammo (Wintersinger and Wasmuth, 2015).

### 2.8. Promoter activity assay

A strain was constructed in which the upstream portion of the gene to be examined was inserted upstream of the *lacZ* gene on the chromosome so that the start codon of the gene exactly matched the start codon of the *lacZ* gene (Supplementary Figure S2). Overnight cultures in LB medium were diluted 1/100 in fresh LB with supplements. Cultures were incubated in test tubes for 3 h at 37°C with shaking (130 r.p.m.). β-galactosidase activities were measured as previously described (Miller, 1972).

### 2.9. Phenylpropionate transporter colorimetric assay

Overnight cultures in LB medium were diluted 1/100 in fresh LB containing 1 mM 3-phenylpropionate. Cultures were incubated in test tubes for 24 h at 37°C with shaking (130 r.p.m.). Cells were subsequently pelleted by centrifugation, and the absorbance of the supernatant was measured at 500 nm.

### 2.10. Competition assay

Mutant and control strains were incubated overnight at 37°C until stationary phase and were then combined in a 1:1 ratio. The presence of equal numbers of viable bacteria of each strain was confirmed using a spot test. Mixed cultures were subsequently diluted 1/100,000 and incubated at 37°C with or without menadione. After 1, 3, and 5 days, the number of viable bacteria was enumerated by diluting samples 1/50, 1/2,500, and 1/125,000; spotting them on AM3 plates; and culturing them at 37°C.

### 2.11. Statistical analysis

Normality and variances in each dataset were determined *a priori* using the Shapiro–Wilk or Bartlett tests. Welch’s *t*-test was used to analyze differences between two groups. An analysis of variance (ANOVA) test was used to analyze differences between three groups, followed by a Tukey’s multiple comparison correction. A Monte Carlo simulation with 100,000 replicates was used for the chi-square test for goodness of fit. *p*-values smaller than 0.05 were considered significant.

## 3. Results and discussion

### 3.1. Construction of genome-reduced strain Δ33b–Δ41c

Previously constructed genome-reduced strains did not lack essential genes but had attenuated growth compared with the WT (Hashimoto et al., 2005). The doubling time of the genome-reduced Δ33a strain, which lacks approximately 38.9% of the WT chromosome, was 170.0 min (Hashimoto et al., 2005, Table 1). To construct genome-reduced strains with further chromosomal deletions, we first attempted to isolate strains with enhanced growth by ALE. After sequentially culturing the Δ33a parent strain for approximately 1,800 generations, we isolated a faster growing variant, termed strain Δ33b, with a doubling time of 113.3 min (Table 1).

**Table 1:** Doubling time of genome-reduced strains.

Strain	Doubling time (min)	SE
Δ33a	170.0	9.4
Δ33b	113.3	7.2
Δ37b	133.3	7.2
Δ37c13	53.3	2.7
Δ37c16	40.0	0.0
Δ37c143	86.7	5.4
Δ37c145	43.3	2.7
Δ37c146	50.0	0.0
Δ41c	136.7	29.9

**Figure 1:**
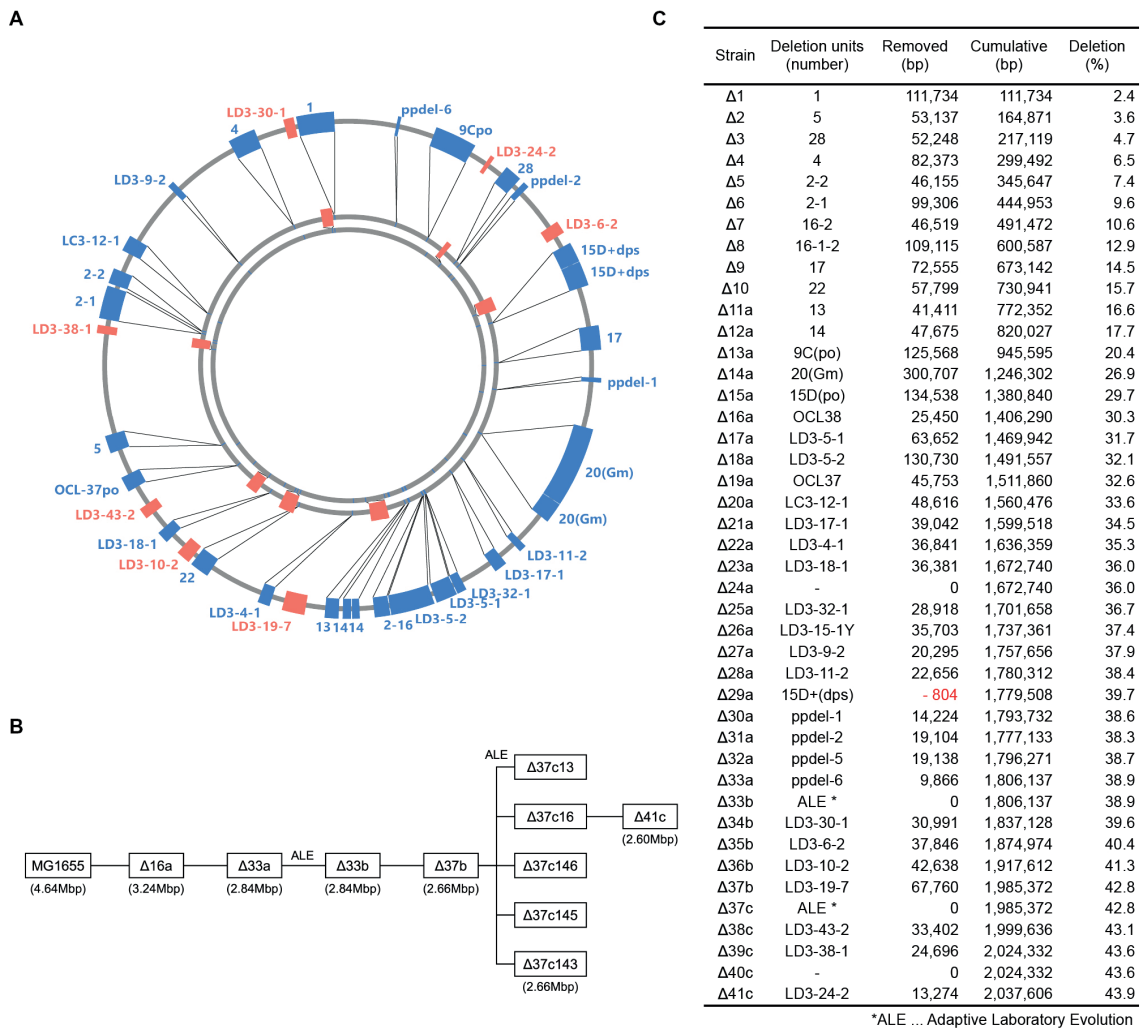
Construction of genome-reduced strains. **(A)** Genetic units deleted in the construction of genome-reduced strains. The genomes of Δ41c, Δ33a, and MG1655 are represented from the center of the circle, respectively. The closed boxes indicate deleted regions. **(B)** Schematic depicting the relationship between constructed genome-reduced strains. **(C)** Table describing the number and length of deletion units.

By introducing additional nonessential chromosomal deletions into strain Δ33b, we constructed additional genome-reduced strains (Δ33b to Δ37b; Figures 1A–C). To sequentially introduce chromosomal deletions, we optimized the FRT4 system to remove positive selection markers (Iwadate et al., 2011, Supplementary Figure S1). First, several large-scale deletions were introduced to intergenic regions between essential genes of the Δ33b strain in the MG1655 *red* strain using an Ap resistance gene. Next, these new deletion mutations were introduced into genome-reduced strains using P1 transduction, and mutations that did not significantly attenuate growth were selected. The positive selection marker was subsequently exchanged with a Cm resistance gene with a FLP-FRT recombination site in the MG1655 *red* strain. Chromosomal regions on both sides of the deletion, in addition to the negative selection marker gene *rpsL*+, and the Ap resistance gene were cloned into a suicide vector containing FRT, and the plasmid was inserted into the chromosome by homologous recombination. Lastly, deletion mutations were introduced into genome-reduced strains using P1 transduction and FLP-FRT site-specific recombination after introduction of a plasmid expressing the FLP recombinase. Sm-resistant, Cm-, and Ap-sensitive strains were then isolated, and the introduction of the deletion was confirmed by PCR.

The doubling time of the genome-reduced strain Δ37b was 133.3 min, longer than that of strain Δ33b (Table 1). To construct genome-reduced strains with further deletion, we first isolated strains with enhanced growth by ALE by performing five independent subcultures, allowing for approximately 1,960–2,300 generations. Following this process, we isolated strains Δ37c-13, 16, 143, 145, and 146 with doubling times of 53.3, 40.0, 86.7, 43.3, and 50.0 min, respectively (Table 1). We next introduced large-scale chromosome deletions into strain Δ37c-16 to generate Δ41c, which harbors deletion of approximately 44% (~2 Mb) of the wild-type chromosome (Figures 1A–C) and has a doubling time of 136.7 min (Table 1).

We next subjected nine genome-reduced strains (Δ33a, the ALE strain Δ33b, Δ37b, the five ALE strains Δ37c, and Δ41c) to whole genome sequencing. We first performed sequencing with the Illumina MiSeq system, followed by long-read sequencing using the MinION system, and identified several SNVs, insertions, and deletions (Table 2; Supplementary Table S1). SNVs, insertions, and deletions were already present in strain Δ33a, indicating that mutations had occurred during construction of the genome-reduced strains. When we examined the types of mutation present in the nine strains, we found that missense and synonymous mutations were most common (Supplementary Figure S3). Insertions, deletions, and genomic rearrangements occurred in all strains, but we found no evidence of common mutational hotspots (Supplementary Figure S4). Genome-reduced strains have previously been identified by the length of the deleted region and the presence or absence of genes, but it is also necessary to classify them based on genomic alterations.

**Table 2:** Mutations identified by genomic resequencing.

		Δ33a	Δ33b	Δ37b	Δ37c13	Δ37c16	Δ37c143	Δ37c145	Δ37c146	Δ41c
SNV	Missense	39	51	59	108	143	114	122	87	159	Synonymous	14	17	17	54	69	53	63	42	83	Frameshift	2	3	1	11	11	4	7	3	22	Stop gained	3	5	6	8	19	8	11	9	21	Stop lost	1	1	1	1	1	1	1	1	2	Upstream	3	4	8	20	16	16	18	17	17	RNA	2	2	1	2	1	2	3	4	1	Intergene	14	14	14	23	26	23	19	20	35
	SNV Total	78	97	107	227	286	221	244	183	340
Insertion		1	3	4	4	4	5	4	4	5
Deletion		1	1	2	3	5	3	4	4	6
Total		80	101	113	234	295	229	252	191	351

### 3.2. Genome alteration from Δ33a to Δ33b

In strain Δ33a, 78 SNVs, 1 insertion, and 1 deletion were identified, whereas in strain Δ33b isolated by ALE, we found 97 SNVs, 3 insertions, and 1 deletion (Table 2; Supplementary Table S1). This indicates that 19 SNVs and 2 insertions occurred during the process of ALE (Table 2; Supplementary Table S1). Some SNVs occurred in essential genes. Most SNVs in essential genes were missense mutations, with the exception of a nonsense mutation in *yceQ* that introduced a stop codon at amino acid position 8. A previous study using transposon mutagenesis reported that *yceQ* itself was not an essential gene but rather contained a promoter for the essential gene *rne* (Goodall et al., 2018). Therefore, all SNVs that occurred in essential genes were missense mutations. If all genes were mutated randomly, we would expect the ratio of mutations in essential genes to nonessential genes to decrease as mutations in essential genes would include lethal mutations. However, in strains Δ33a and Δ33b, we found a significantly higher proportion of mutations in essential genes (Supplementary Table S2). These results suggest that during the process of constructing strain Δ33a and that of isolating strain Δ33b by ALE, mutations in essential genes may have enhanced growth.

**Figure 2:**
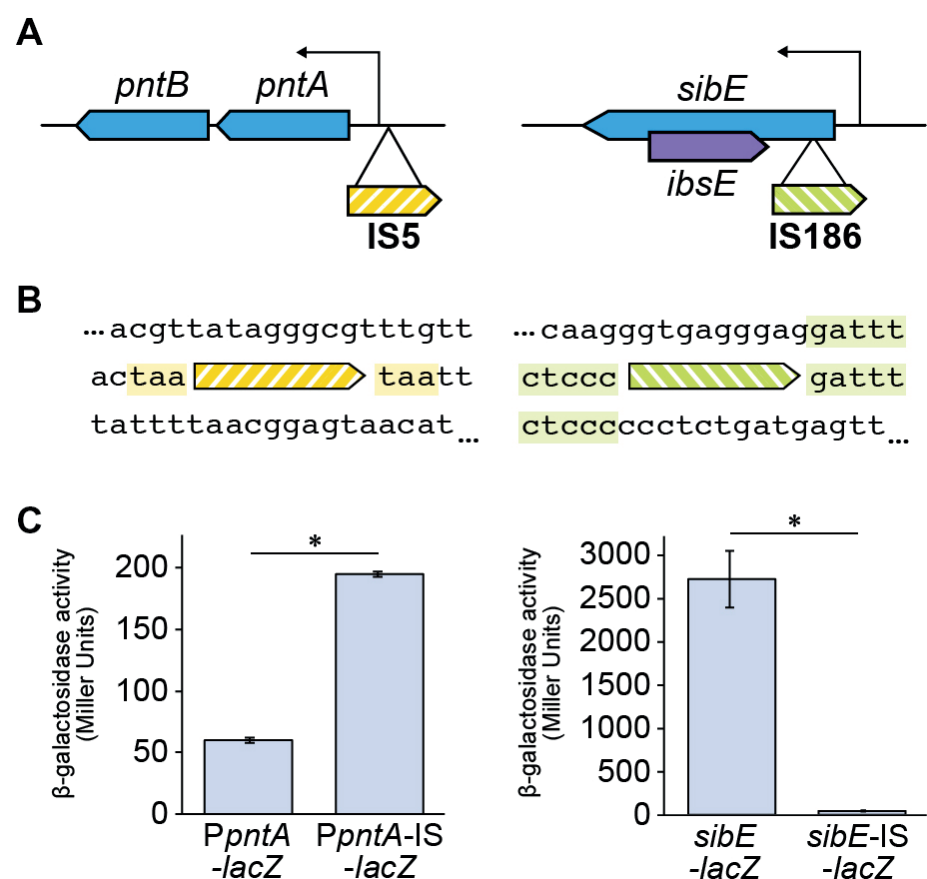
Insertions identified in strain Δ33b. **(A)** Schematic depicting the location of the identified insertions. **(B)** Alignment showing the sequences adjacent to the identified insertions. **(C)** Bar chart depicting the effects of the identified insertions on gene expression. The bar chart shows the mean ± standard error values (*n* = 3). The insertion significantly increased the expression from the *pntA* promoter (*p* < 0.01), while the expression of *sibE* was significantly decreased (*p* = 0.021).

We found two IS insertion mutations within the *pntA* promoter region and the *sibE* gene (Figures 2A,B). Using a *lacZ* reporter gene, we found that the expression of *pntA* was increased by the promoter insertion (Figure 2C). We also found that the *sibE* insertion decreased expression of the gene (Figure 2C). PntA is a proton transporter that synthesizes NADPH from NADH by proton influx (Clarke and Bragg, 1985). SibE, which produces a small RNA, is the antitoxin component of a toxin-antitoxin system and suppresses expression of the toxin gene *ibsE*, which exists in the form of reverse overlap within the *sibE* gene (Fozo et al., 2008). There are five chromosomal *ibs* homologues (ibsA, B, C, D, E) that encode putative membrane proteins. Although their exact functions are unknown, their overexpression is known to cause depolarization (Fozo et al., 2008). Both of the two insertions identified in Δ33b are distinct from simply destructive insertions. This suggests that there is a functional benefit conferred by the insertion that may pertain to growth rate. One potential mechanism for the insertion-mediated rescue of growth is that antitoxin is diminished by the *sibE* insertion, causing overexpression of the IbsE toxin, which subsequently increases membrane potential. Furthermore, insertion-mediated overexpression of PntA may increase levels of NADPH, promoting growth or resistance to oxidative stress.

### 3.3. Genome alterations in Δ37c relative to Δ37b

**Figure 3:**
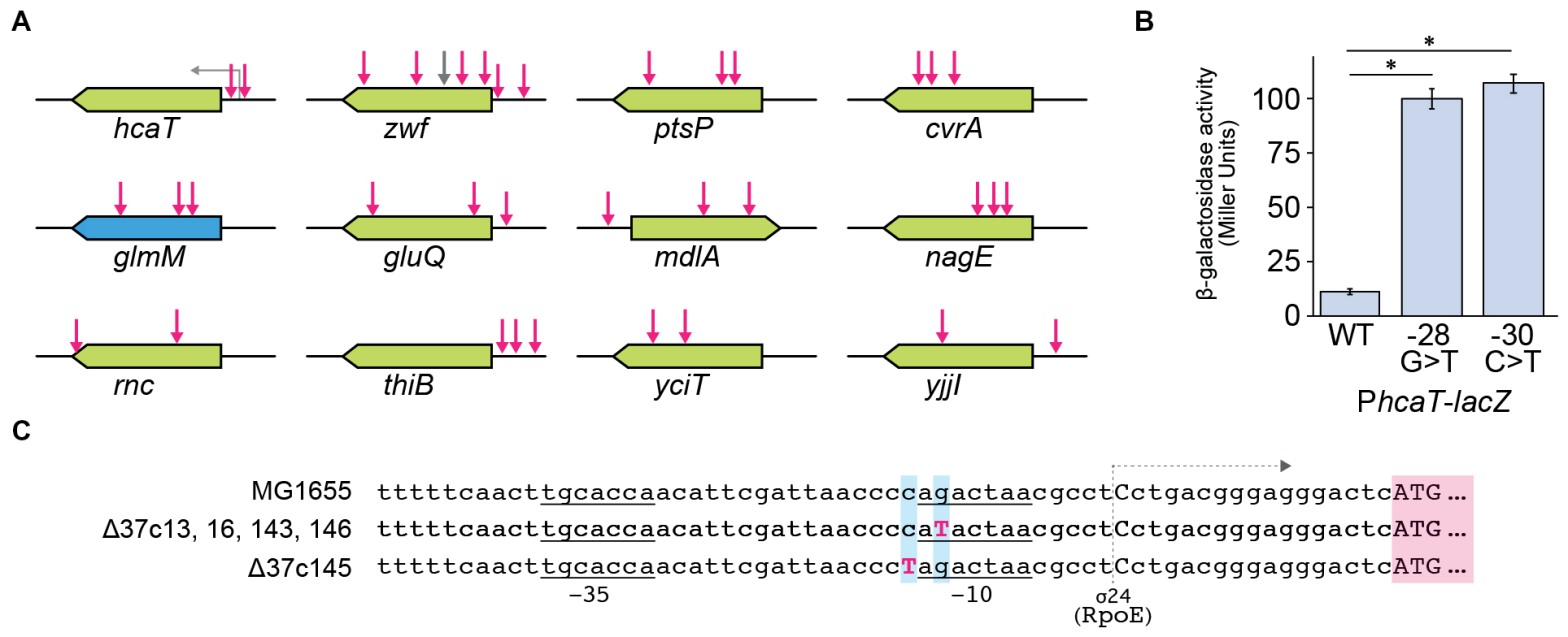
SNVs common to Δ37c ALE strains. **(A)** Schematic depicting mutation sites. Green boxes indicate nonessential genes, and blue boxes indicate essential genes. Red arrows indicate the location of mutations. **(B)** Bar chart depicting the effects of *hcaT* mutations on gene expression. The bar chart shows the mean ± standard error values (*n* = 3). Both mutations significantly increased gene expression (*p* < 0.01). Numbers indicate base positions calculated relative to the transcription start site as +1. **(C)** Sequence alignment of identified *hcaT* mutations. −10 and −35 indicate promoter regions for sigma 24, and gray arrows represent transcription.

**Figure 4:**
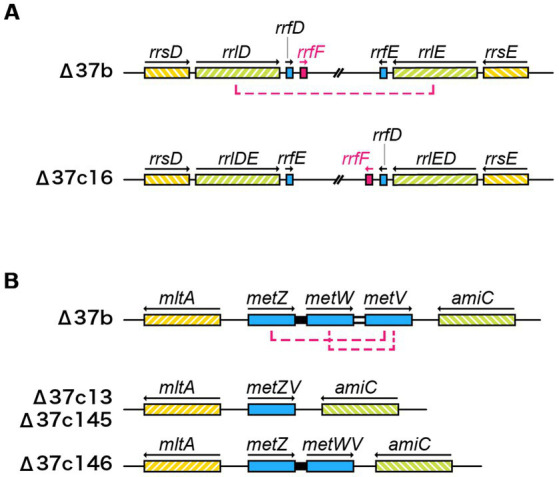
Inversions and deletions identified in Δ37c ALE strains. **(A)** Schematic showing the inversion between *rrnD* and *rrnE* in strain Δ37c16. **(B)** Deletion in the initiating methionine tRNA gene cluster. Two initiating methionine tRNAs (Δ37c13, 145) or one initiating methionine tRNA (Δ37c146) were deleted from the *metZWV* region by homologous recombination.

In strain Δ37b, we identified 107 SNVs, 4 insertions, and 2 deletions. Furthermore, we identified increased SNVs, insertions, and deletions in five Δ37c strains (Table 2; Supplementary Tables S1, S3). Since the five Δ37c strains were isolated independently, we predicted that mutations common to all strains underpin the rescued growth phenotype. We identified 12 genes with SNVs present in 2 or more strains (Figure 3A; Supplementary Table S3). Interestingly, the *hcaT* gene contained SNV in all five strains (Figure 3A; Supplementary Table S3). Furthermore, all SNVs were present upstream of *hcaT*, most commonly in the −10 region of the promoter (Figures 3A,C). Using a LacZ reporter assay, we found that all SNVs increased gene expression (Figure 3B). We also found SNVs in the region upstream of the *thiB* gene, but these mutations did not increase expression except for 37c145 (Supplementary Figure S5). In addition to SNV, we identified an inversion between *rrn* loci in one out of five strains (Figure 4A), and deletions in f-Met tRNA (Figure 4B). Three tandem copies of f-Met tRNA are present on the wild-type chromosome, and we observed deletion of one or two copies through homologous recombination (Figure 4B). Although three out of five strains showed reduced copy number, it remains unclear whether this altered growth of the genome-reduced strains.

### 3.4. Analysis of *hca* and *mhp* mutant strains

**Figure 5:**
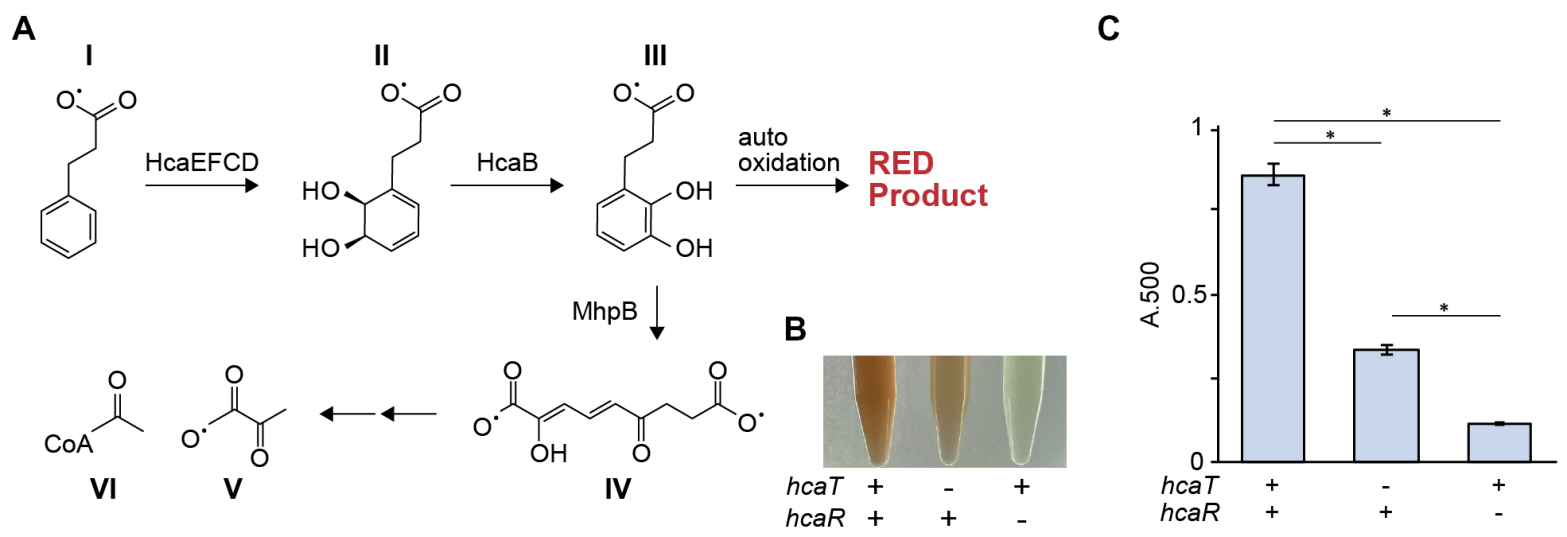
*hca* genes are involved in phenylpropionate degradation. **(A)** The phenylpropionic acid (I) degradation pathway. Phenylpropionate is degraded to succinate (V) and acetyl CoA (VI) *via* several metabolic intermediates (II-IV). **(B)** Color of culture supernatants after growth of *ΔmhpR-T*, *ΔmhpR*-*T ΔhcaT*, and *ΔmhpR-T ΔhcaR* mutants in the presence of phenylpropionate. **(C)** Absorbance of culture supernatants at OD500 nm. The bar chart shows the mean ± standard error of absorbance measurements (*n* = 3, Δ*mhpRT* is *n* = 4). The deletion of *hcaT* significantly suppressed pigmentation of the medium, but the color change was still observed even in the presence of *hcaT* deletion (*p* < 0.01).

Although *hcaT* was implicated in the growth rescue of strain Δ37b, the function of this gene remains poorly understood. We constructed and phenotypically characterized deletion strains lacking *hcaT*, *hcaR*, and *hcaE*-*hcaD* genes, as well as *mhp* related genes. The sequence of HcaT indicates this protein is a putative transporter, but its substrate is not known. The *hcaE*-*hcaD* and *mhp* gene clusters are encoded close to *hcaT* and are involved in acetyl-CoA synthesis by breaking down 3-phenylpropionate (Pao et al., 1998; Díaz et al., 2001). Since *hcaR*, which is encoded immediately upstream of *hcaT*, is an activator of the *hca* gene cluster, *hcaT* has also been speculated as a 3-phenylpropionate transporter (Díaz et al., 1998). Auto-oxidation of 3-(2,3-dihydroxyphenyl) propionate, which accumulates when the *mhp* gene cluster is deleted, oxidizes proline in the growth medium, causing a color change to red (Burlingame et al., 1986) (Figure 5A). Double mutant strains were constructed lacking the *mhp* gene cluster and either *hcaT* or *hcaR*. The *mhp hcaR* double mutant did not cause pigmentation of the medium although the *mhp hcaT* mutant showed redder medium than the *mhp hcaR* double mutant but not as much as the *mhp* single mutant (Figures 5B,C). Since pigmentation of the medium does not occur unless 3-phenylpropionate is present, these results suggest that HcaT is a 3-phenylpropionate transporter (Figures 5B,C; Supplementary Figure S6). In addition, we examined the growth of constructed strains using 3-phenylpropionate as a carbon source. On minimal medium supplemented with 3-phenylpropionate, wild-type strains were able to grow, but *hcaR*, *hcaE*-*hcaD*, *mhp*T-*mhp*R, and *mhp*T-*mhp*R *hcaT* deletion strains were not (Supplementary Figure S7). Since *hcaT* deletion mutants were able to grow, these results also suggest the presence of additional 3-phenylpropionate transporters other than HcaT.

3-phenylpropionate is considered a quality control measure during protein synthesis. 3-phenylpropionate competitively inhibits the ligation of phenylalanine to tRNA^Phe^ by phenylalanine aminoacyl-tRNA synthetase (PheRS) (Mulivor and Rappaport, 1973). Tyrosine can be erroneously ligated to tRNA^Phe^ by PheRS but is subsequently hydrolyzed and removed by the proofreading activity of PheRS (Roy et al., 2004). Cytotoxic meta-tyrosine is generated from tyrosine in the presence of oxidative stress (Bullwinkle et al., 2014). The fact that the proofreading activity of PheRS increases under oxidative stress suggests an important role for the removal of meta-tyrosine (Steiner et al., 2019). In strain Δ37c, in addition to deletion of the *mhp* gene cluster, we predict that levels of HcaT increase due to mutation of the *hcaT* promoter, which subsequently increases the levels of intracellular 3-phenylpropionate. 3-phenylpropionate may subsequently reduce levels of meta-tyrosine-tRNA^Phe^, improving the quality of protein synthesis and restoring growth.

**Figure 6:**
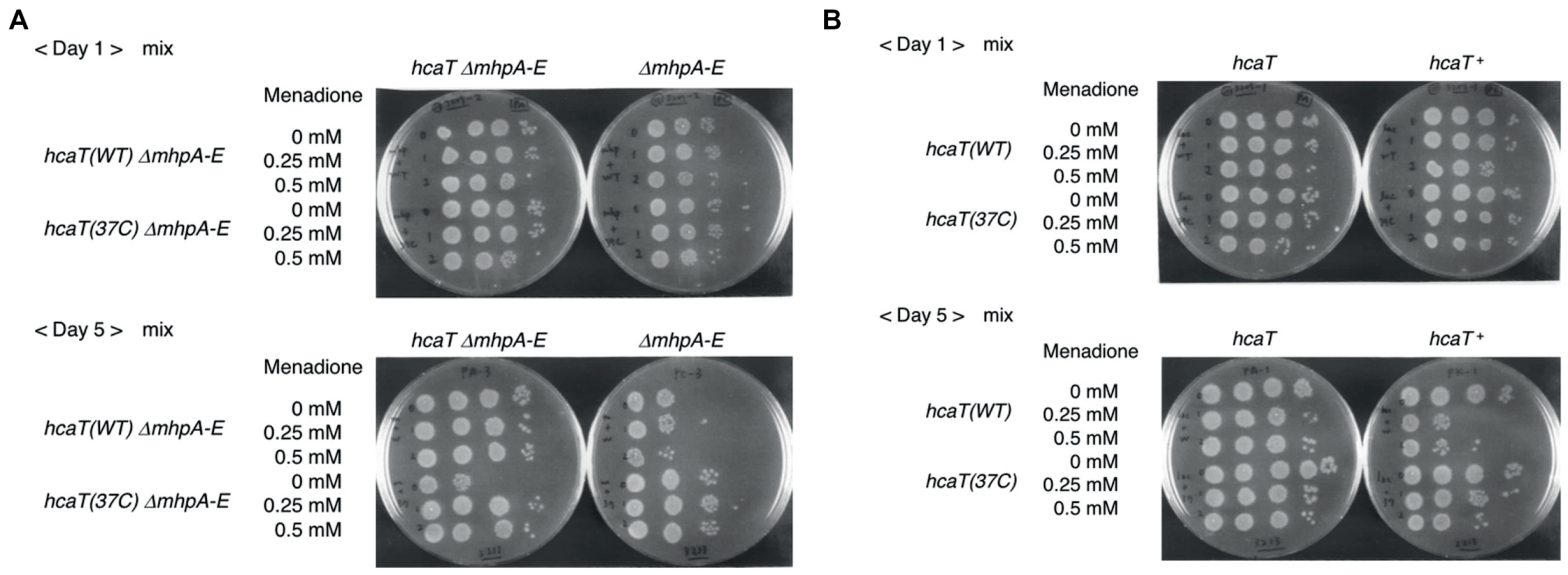
Competition assay with the strain harboring the *hcaT* mutation identified in Δ37c-16. **(A)** Double mutant, *hcaT* (37c) Δ*mhpAE*, and control, *hcaT* (WT) Δ*mhpAE*. Spotting cultures are shown after day 1 and day 5 of growth. **(B)** Mutant, *hcaT* (37c) and control, *hcaT* (WT) are shown after day 1 and day 5 of growth.

HcaR is involved in expression of scavenger enzymes, which remove reactive oxygen species, and has been associated with the oxidative stress response (Turlin et al., 2005). Since the *mhp* gene cluster is deleted in strain Δ37b, we constructed a double mutant harboring both a *mhp* deletion and a SNV mutation (*hcaT* (37c)) that increases *hcaT* expression. We investigated the survival of long-term stationary phase cultures in the presence of oxidative stress and the redox-cycling drug menadione. When the *mhp* deletion mutant and the *mhp hcaT* (37c) double mutant strain were cultured alone, we observed no significant difference in growth (Supplementary Figure S8). However, when those strains were cultured together for 5 days, the *mhp* mutant showed enhanced survival (Figure 6A). The same results were also obtained in the *mhp* + background, albeit to a lesser extent (Figure 6B). Survival of not only of the *mhp hcaT* (37c) double mutant but also the mixed *mhp* deletion strain suggested a change to the composition of the medium. Increased expression of *hcaT* in the *mhp hcaT* (37c) double mutant may change the composition of the medium by taking up and metabolizing 3-phenylpropionate, promoting survival of the strain in the presence of oxidative stress at least in the conditions where a large number of genes were deleted like the genome-reduced strain constructed in this work. Further characterization of the functions of 3-phenylpropionate and related compounds will elucidate the mechanism underpinning this phenotype.

A recent study demonstrated rewiring of imbalanced metabolism in genome-reduced strains through isolation and analysis of strains with growth restored by ALE (Choe et al., 2019). This may enable characterization of the stress resistance mechanism, which promotes survival in stationary phase. Analysis of genome-reduced strains in which the various systems are imbalanced as a resource reveals aspects that are different from research with wild-type strains and individual gene deletion mutants. These studies will shed fundamental insights into cell proliferation and survival.

## Data availability statement

The datasets presented in this study have been deposited in the DNA Data Bank of Japan, accession number PRJDB15441: https://ddbj.nig.ac.jp/resource/bioproject/PRJDB15441.

## Author contributions

YK, MH, KL, and JK designed the study and performed the experiment. YK analyzed and visualized the data. JK supervised the study. YK and JK wrote and revised the manuscript. All authors contributed to the article and approved the submitted version.

## Funding

This work was supported by Grants of Tokyo Metropolitan University to JK.

## Conflict of interest

The authors declare that the research was conducted in the absence of any commercial or financial relationships that could be construed as a potential conflict of interest.

## Publisher’s note

All claims expressed in this article are solely those of the authors and do not necessarily represent those of their affiliated organizations, or those of the publisher, the editors and the reviewers. Any product that may be evaluated in this article, or claim that may be made by its manufacturer, is not guaranteed or endorsed by the publisher.
