# Supplementary material for: Mutations identified in engineered Escherichia coli with a reduced genome
Source: Front Microbiol. 2023 May 25;14:1189877. doi: 10.3389/fmicb.2023.1189877 (PMC10249474; doi:10.3389/fmicb.2023.1189877)
Supplement: Supplementary file 4 [file Data_Sheet_1.PDF]

Supplementary Material

Mutations identified in engineered Escherichia coli with a reduced genome

Yuto Kotaka[1,2], Masayuki Hashimoto[3], Ken-ichi Lee[2], Jun-ichi Kato[1*]

*** Correspondence:** Jun-ichi Kato: jkato@tmu.ac.jp

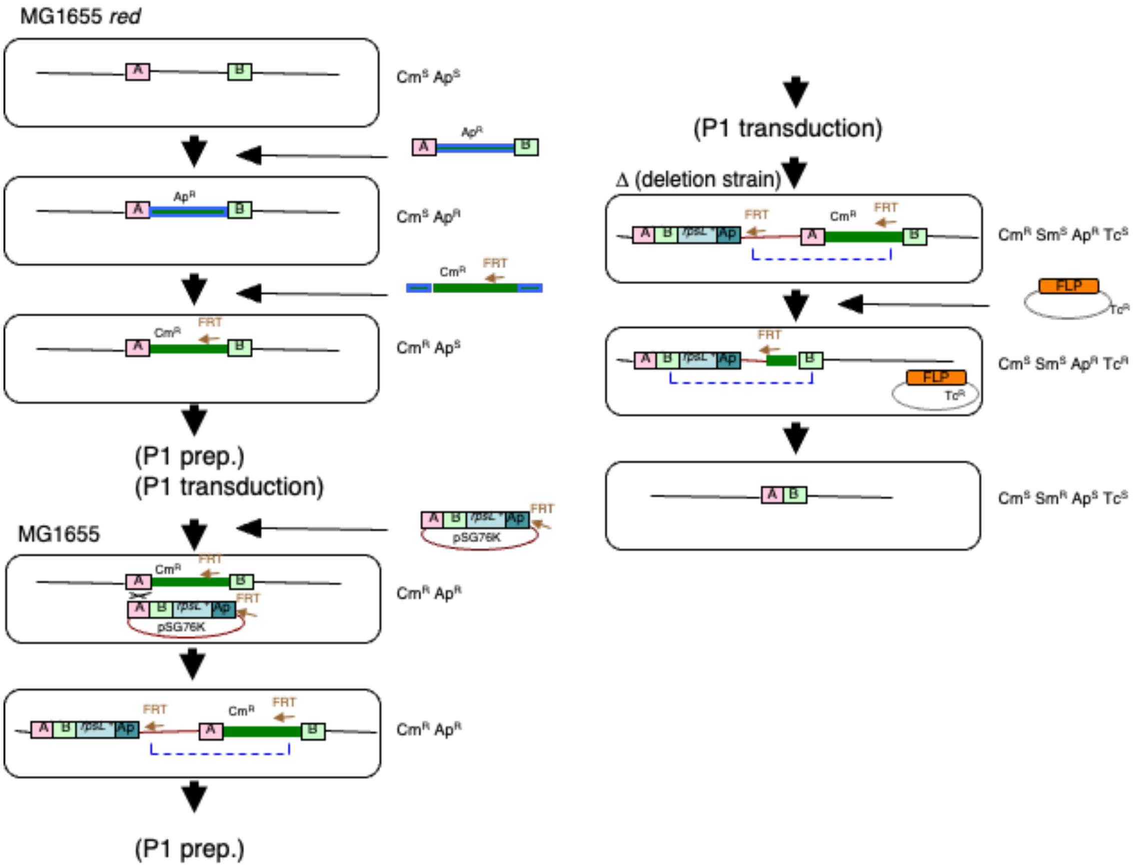

Supplementary Figure 1.

A method for generating large markerless chromosome deletions to construct genome-reduced strains. The FRT4 system was improved as described in the Materials and methods and Results and discussion sections (Iwadate et al., 2011).

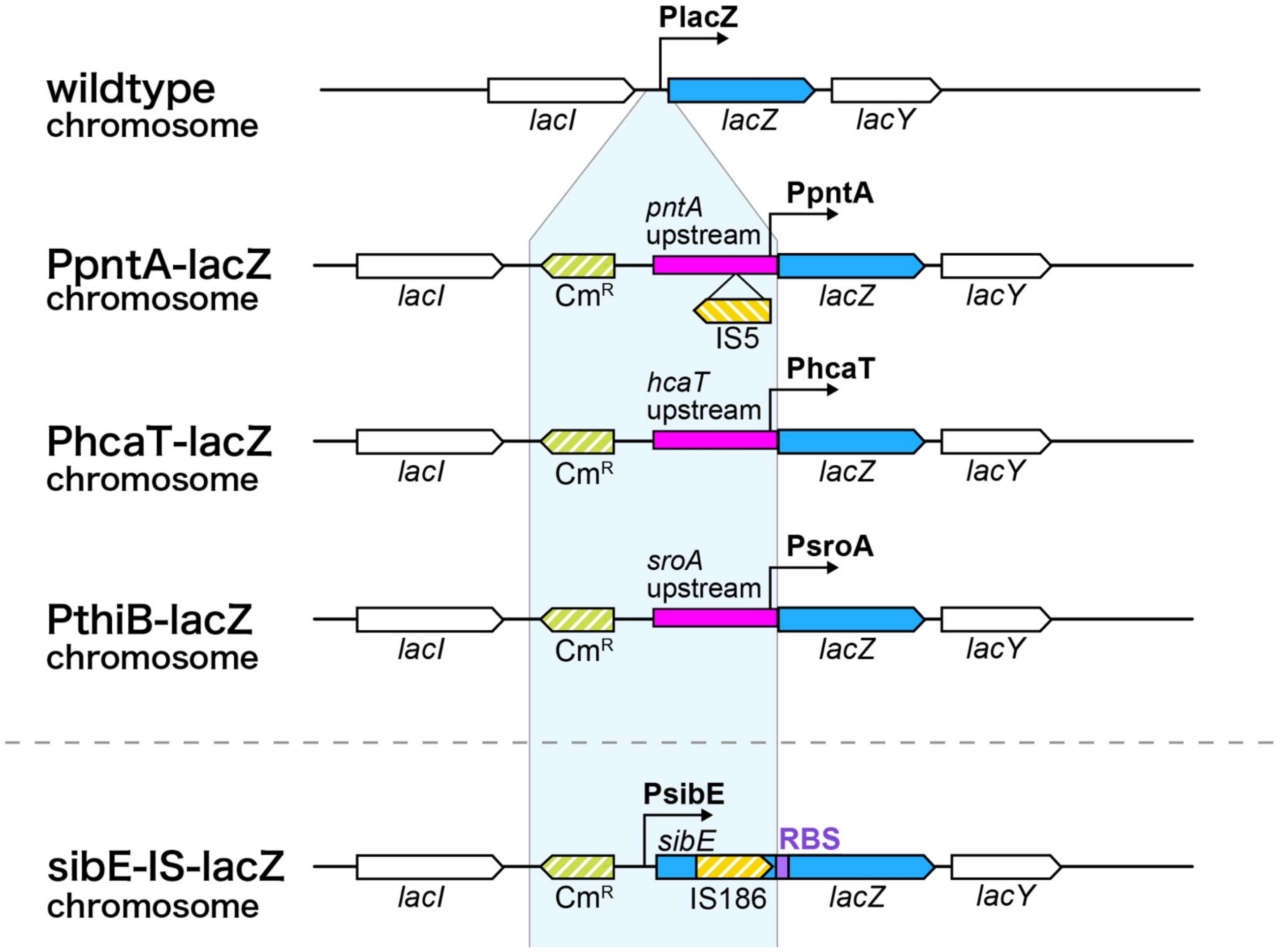

Supplementary Figure 2.

Constructs used for the β-galactosidase assay. The constructs were made by linking the upstream region of each gene to the *lacZ* locus of the chromosome.

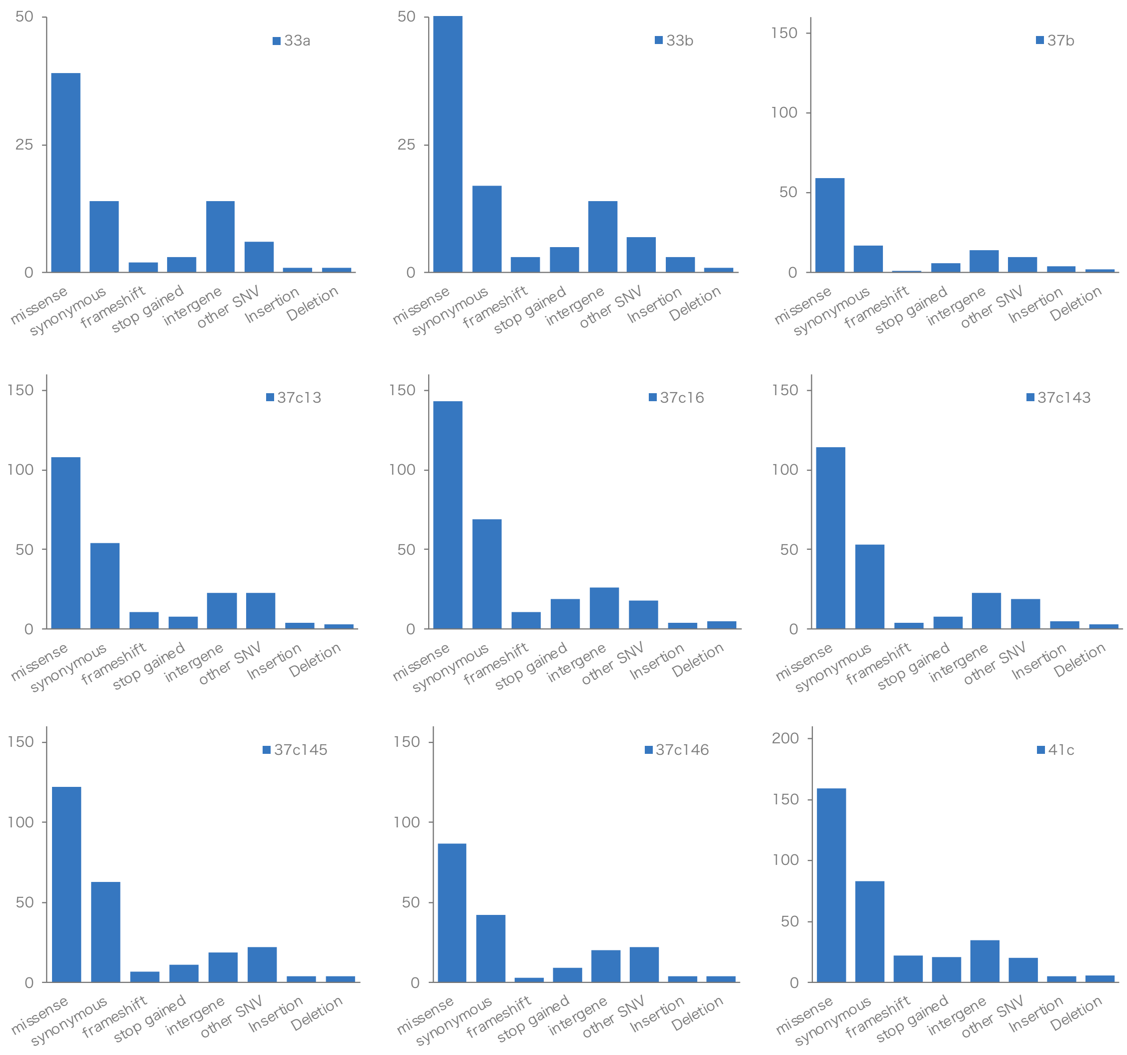

Supplementary Figure 3.

Mutations identified in genome-reduced strains.

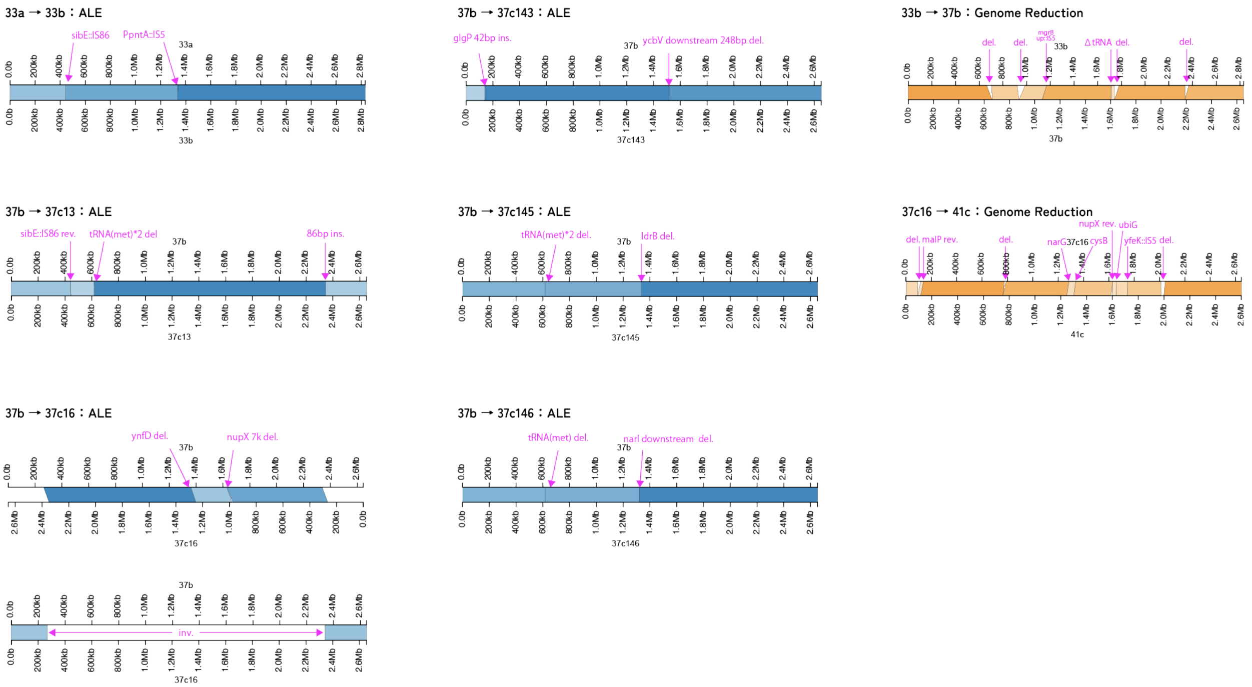

Supplementary Figure 4.

Schematic depicting genomic DNA rearrangements identified by BLAST and Kablammo in constructed strains. Blue- or orange-colored regions indicate high levels of homology.

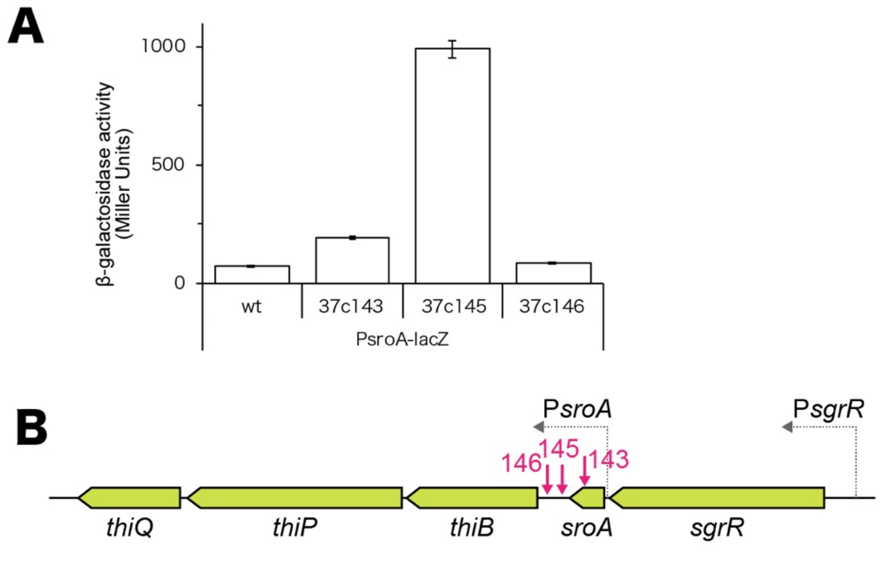

Supplementary Figure 5.

common mutation to *thiB* of Δ37c ALE strains. (A) Bar chart depicting the effects of *thiB* mutations on gene expression. The bar chart shows the mean ± standard error values (n = 3). (B) Schematic depicting mutation sites.

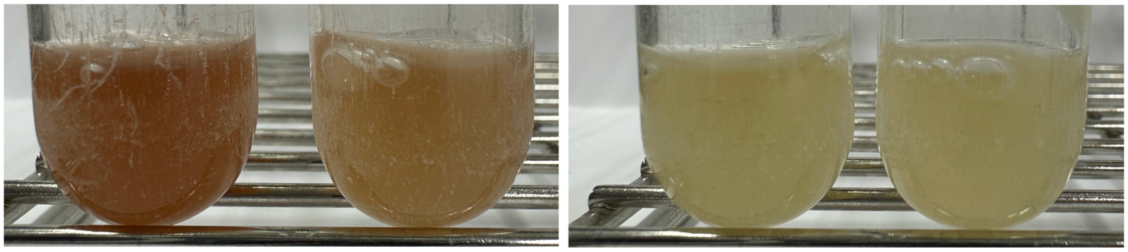

3-PP	+	+	-	-
mhp	-	-	-	-
hcaT	+	-	+	-

Supplementary Figure 6.

Cultured *mhp* strain under the same conditions in LB medium with/without 3-phenylpropionate (3-PP). Pigmentation of the medium does not occur unless 3-phenylpropionate is present.

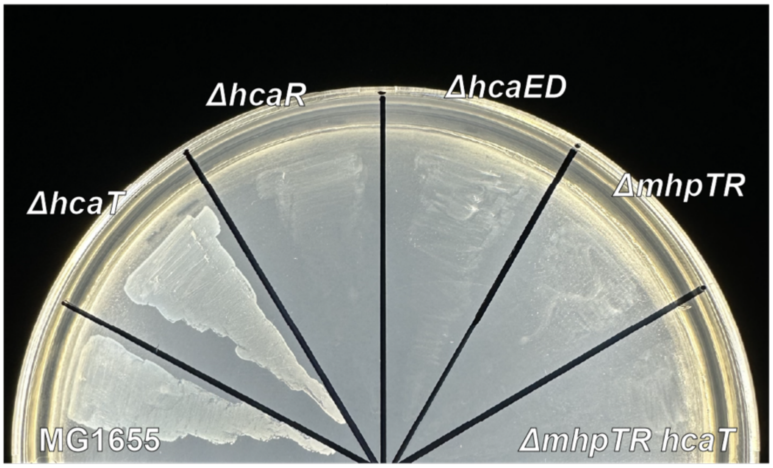

Supplementary Figure 7.

Growth of constructed mutants using 3-phenylpropionate as a carbon source. Wild-type and *hca* and *mhp* deletion mutants were grown on M9 plate supplemented with 3-phenylpropionate at 37℃.

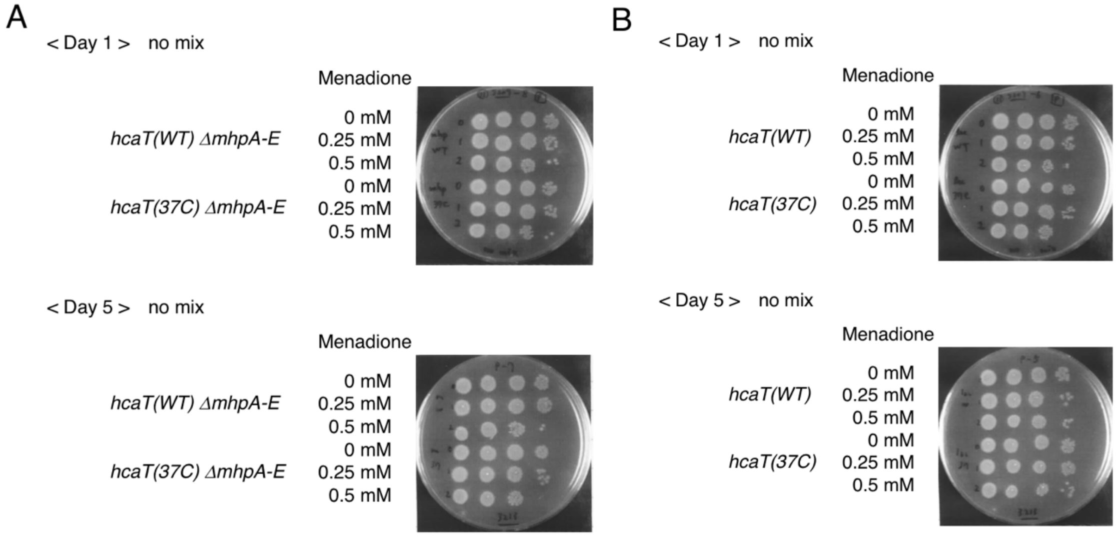

Supplementary Figure 8.

Spot assay using the strain harboring the *hcaT* mutation identified in Δ37c-16. (A) Double mutant, *hcaT* (37c) Δm*hpAE*, and control, *hcaT* (WT) Δ*mhpAE*. At day 1 and day 5 of growth. (B) Mutant, *hcaT* (37c) and control, *hcaT* (WT) at day 1 and day 5 of growth.
